# Improving predictive asthma algorithms with modelled environment data for Scotland: an observational cohort study protocol

**DOI:** 10.1136/bmjopen-2018-023289

**Published:** 2018-05-20

**Authors:** Ireneous N Soyiri, Aziz Sheikh, Stefan Reis, Kimberly Kavanagh, Massimo Vieno, Tom Clemens, Edward J Carnell, Jiafeng Pan, Abby King, Rachel C Beck, Hester J T Ward, Chris Dibben, Chris Robertson, Colin R Simpson

**Affiliations:** 1 Asthma UK Centre for Applied Research, Usher Institute of Population Health Sciences and Informatics, Centre for Medical Informatics, The University of Edinburgh, Edinburgh, UK; 2 Atmospheric Chemistry and Effects, NERC Centre for Ecology & Hydrology, Penicuik, UK; 3 Knowledge Spa, University of Exeter Medical School, Truro, UK; 4 Department of Mathematics and Statistics, The University of Strathclyde, Glasgow, UK; 5 School of Geosciences, Institute of Geography, The University of Edinburgh, Edinburgh, UK; 6 Information Services Division and Health Protection Scotland, NHS National Services Scotland, Edinburgh, UK; 7 Faculty of Health, Victoria University of Wellington, Wellington, New Zealand

**Keywords:** asthma, primary care, learning health system, environmental epidemiology, pollution effects

## Abstract

**Introduction:**

Asthma has a considerable, but potentially, avoidable burden on many populations globally. Scotland has some of the poorest health outcomes from asthma. Although ambient pollution, weather changes and sociodemographic factors have been associated with asthma attacks, it remains unclear whether modelled environment data and geospatial information can improve population-based asthma predictive algorithms. We aim to create the afferent loop of a national learning health system for asthma in Scotland. We will investigate the associations between ambient pollution, meteorological, geospatial and sociodemographic factors and asthma attacks.

**Methods and Analysis:**

We will develop and implement a secured data governance and linkage framework to incorporate primary care health data, modelled environment data, geospatial population and sociodemographic data. Data from 75 recruited primary care practices (n=500 000 patients) in Scotland will be used. Modelled environment data on key air pollutants at a horizontal resolution of 5 km×5 km at hourly time steps will be generated using the EMEP4UK atmospheric chemistry transport modelling system for the datazones of the primary care practices’ populations. Scottish population census and education databases will be incorporated into the linkage framework for analysis. We will then undertake a longitudinal retrospective observational analysis. Asthma outcomes include asthma hospitalisations and oral steroid prescriptions. Using a nested case–control study design, associations between all covariates will be measured using conditional logistic regression to account for the matched design and to identify suitable predictors and potential candidate algorithms for an asthma learning health system in Scotland.

Findings from this study will contribute to the development of predictive algorithms for asthma outcomes and be used to form the basis for our learning health system prototype.

**Ethics and dissemination:**

The study received National Health Service Research Ethics Committee approval (16/SS/0130) and also obtained permissions via the Public Benefit and Privacy Panel for Health and Social Care in Scotland to access, collate and use the following data sets: population and housing census for Scotland; Scottish education data via the Scottish Exchange of Data and primary care data from general practice Data Custodians. Analytic code will be made available in the open source GitHub website. The results of this study will be published in international peer reviewed journals.

**Strengths and limitations of this study**

- Development and implementation of a novel data governance and linkage framework to incorporate primary care health, modelled environmental, geospatial population and sociodemographic data.
- Data from 75 recruited primary care practices (n=500 000 patients) in Scotland will be used, 10% Scottish population.
- We will create a daily model forecast of ambient air pollution and meteorology for 72 hours for the study area at a high geospatial resolution and for specific pollutants.
- Like many data sources, the accuracy of modelled environment data (including ambient pollution and meteorological measures) that was generated from an atmospheric chemistry transport modelling system may affect the predictive asthma algorithm.
- We will need to evaluate whether the new model affects care and improves outcomes for patients in future studies.

## Introduction

Scotland has among the highest prevalence of asthma in the world and some of the poorest health outcomes from asthma.^1^ Asthma is, as a result, responsible for considerable—potentially avoidable—morbidity, healthcare utilisation and mortality,^2^ with an estimated cost to Scotland of at least £92 m per annum, over £10 m of which is spent on unscheduled care.^3^ Serial confidential enquiries into UK asthma deaths have consistently highlighted major deficiencies in care and have come to the striking conclusion that around half of asthma deaths per year in the UK are potentially avoidable.^4^ Furthermore, our detailed analysis of Scottish data has highlighted that there has been no decrease in rate of asthma hospitalisations or deaths in Scotland over the last decade.^3^ To help address these poor health outcomes, we are working to develop a learning health system (LHS) for asthma in Scotland, which will allow real-time access to, and interrogation of, a wide range of data sources to provide tailored feedback to healthcare professionals in a format that they can readily action.

Pollution, meteorological, lifestyle and sociodemographic factors are known to contribute to asthma outcomes including morbidity and mortality. These factors by themselves have complex interactions that may mediate their overall impact on asthma outcomes such as asthma attacks. However, there are no comprehensive studies, which have examined the effects of ambient pollution, sociodemographic and lifestyle factors on asthma in large populations. Epidemiological studies have demonstrated the short-term and long-term adverse respiratory/asthma health effects resulting from exposure to pollution and weather conditions such as extremes in temperature and humidity.^5–7^ For instance, school proximity to urban infrastructure (eg, highways and industry) has been linked to poor asthma outcomes.^8–10^ Indoor air pollution (eg, from tobacco smoke or volatile organic compounds), as well as exposure to ambient air pollutants (including fine particles and nitrogen oxides) and pollen have all been shown to be associated with asthma attacks.^6 11–15^ Key asthma care gaps can also lead to asthma attacks, for example, poor clinical guideline adherence^16^ and a lack of guided self-management/provision of personal asthma action plans.^17^ Nonetheless, there has been little work to demonstrate the combined effects of air pollutants, meteorological factors, sociodemographic factors and healthcare gaps on asthma attacks.

The current approaches to risk prediction have been weakened by an inability to include reliable modelled ambient air pollution and meteorological data.^7 18 19^ Previous studies have estimated pollution exposures, for example, particulate matter from monitoring stations, attributing the value at the monitoring station to all locations around it (frequently up to 5–10 km away). This lack of spatial representativeness of existing monitoring network data presents a substantial barrier for the identification of associations between pollution events and health effects.^20^ In addition, the use of only residential location has meant that the spatiotemporal representativeness of measured air pollutant concentrations for the estimation of population exposure has been suboptimal.^21 22^

The specific challenge of predicting individuals likely to have an asthma attack who require clinical care can be addressed through the interrogation and analysis of general practice and other healthcare data linked to data sources such as meteorological, pollution, school and census data. Asthma represents an appropriate exemplar condition to investigate the value of bringing these data assets together because it is very common, affects people of all ages, both genders and urban and rural populations, and because pollution and weather are both important risk factors for asthma attacks. We aim to investigate the associations between ambient pollution, meteorological, geospatial and sociodemographic factors, which will aid the future development of predictive algorithms for asthma outcomes.

Our objectives are to:

1. Gain the necessary permissions required to compile and use longitudinal disparate data sets from clinical and health services databases, environment, geospatial, population and sociodemographic and education databases.
2. Model potential meteorological and pollution predictors of asthma at higher resolutions.
3. Investigate geospatial locations and mobility, exposure at work place, schools and residence.
4. Understand the associations between environmental and health covariates and clinically relevant asthma outcomes.
5. Investigate the feasibility of creating a daily pollution and meteorological forecast for use in an operational learning health system.

## Methods

### Patient and public involvement (PPI)

Patients and or the public were not involved in the development of the research question and outcome measures of this study; however, the PPI group of the Asthma UK Centre for Applied Research (AUKCAR) commented on the early design of the LHS for asthma in Scotland project. Patients were not involved in the recruitment and conduct of the study. We plan to use the AUKCAR’s Patient and Public Involvement Platform in the dissemination of our results (www.aukcar.ac.uk/public-involvement).

### Study design, data sources and study period

**Figure 1:**
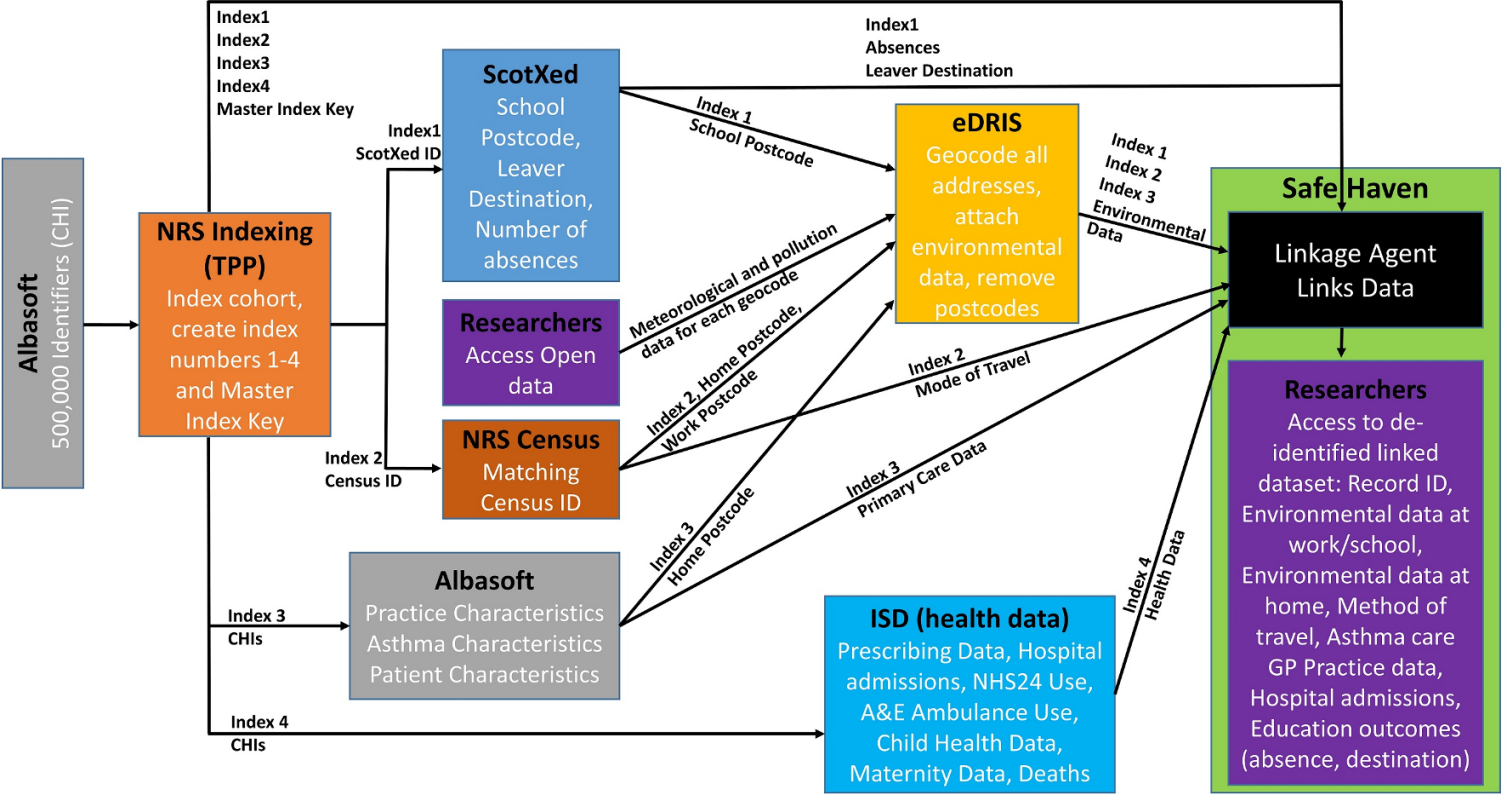
Data linkage framework in a secured environment.

A longitudinal retrospective observational study, involving at least two nested case–control studies, using a national primary care linked database will be carried out. These studies will inform our further studies. The period of analysis will range from 1 January 2013 to 31 December 2015. The respective health (including key outcomes), environment, education and census data sets that will act as the exposure variables and confounding factors/effect modifiers are described subsequently, and the process of data linkage is illustrated in figure 1.

### Health data

Almost all individuals resident in Scotland are registered with a general practice, which provides and coordinates healthcare services, including prescriptions, free of charge. We will use data from about 500 000 patients from 75 primary care practices from across Scotland. The main health outcomes of interest are daily asthma hospitalisations and daily oral steroid prescriptions. These variables, together with other patient characteristics such as sex, body mass index and previous attacks, which are linked to the encrypted unique patient identifier—the Community Health Index number (CHI), will be extracted. The anonymised primary care data will be linked (using CHI) to the national unscheduled care DataMart, which includes Accident and Emergency (emergency room known as A&E in the UK) and ambulance data and hospital/mortality data sets (held by the Information Services Division for the purpose of linkage and research).

### Environmental data

We will use the EMEP4UK atmospheric chemistry transport modelling system (http://www.emep4uk.ceh.ac.uk/), which comprises the state-of-the-art Weather Research and Forecasting (WRF) meteorological model and the EMEP4UK Chemistry Transport Model. EMEP4UK generates geospatially and temporally resolved data on key air pollutants at a horizontal resolution of 5 km×5 km at hourly time steps for nitrogen oxides (NO, NO_2_), ground level ozone (O_3_), sulphur dioxide (SO_2_) and the main components of fine particulate matter (PM_2.5_ and PM_10_) as well as meteorological factors including temperature, wind speed/direction and humidity.

### Census and school data

To enhance our exposure estimation for people with asthma, we will link patient data from primary care practices to the 2011 Scottish national Census and ScotXed, an education database with school attendance at the individual patient level. We will extract information relating to school and work location, hours spent at work, mode of transport taken when travelling to work as well as other individual level socioeconomic and demographic data. This will generate greater contextual information about the patients.^23–25^ We will use Data Zones as our area unit of analysis and calculate exposures at the locations provided by school and census data.

### Data linkage

We will perform data linkage involving cross-sectoral administrative data and environmental and National Health Service (NHS) health data. Available information from pollution, census and education databases will be linked to the individual patient health data (using the individual CHI number and geocodes) to allow us to implement more detailed exposure models by linking to concentration estimates pertaining to residential, work and commuting locations (figure 1).

### Modelling environmental factors

We will use the EMEP4UK atmospheric chemistry transport modelling system (http://www.emep4uk.ceh.ac.uk/) and provide both historic model outputs for a multiyear period to input into a statistical analysis and to develop and evaluate an operational Scotland-wide high resolution (~1 km×1 km) atmospheric composition forecast for priority gaseous air pollutants (nitrogen oxides—NO, NO_2_ and ozone—O_3_) and the main components of PM_2.5_ and PM_10_. The following tasks will be undertaken.

1. Undertake a scoping study for setting up the model framework and input parameters for both the meteorology (Weather Research Forecast model, WRF) and the atmospheric chemistry (atmospheric chemistry transport model, EMEP4UK).
2. Compare model forecasts with hourly observations of air pollutant concentrations from existing regulatory monitoring networks to validate model output at the appropriate scale and resolution.
3. Develop a robust statistical method to test the EMEP4UK model forecast skills and use this information to quantify uncertainties in the model results, which feeds into the final statistical analysis.

### Statistical analysis

For the two outcomes specified—asthma hospitalisations and oral steroid prescriptions—we will construct from the cohort, two nested case–control studies whereby the cases are those with the relevant outcome. As not everyone with asthma may be identifiable (in terms of a risk group coding) from the data prior to an attack, we will include all individuals in the extraction process for cases and the subsequent matching of controls. All cases will be identified with corresponding date of exacerbation dependent on the outcome—either date of hospitalisation or date of prescription dispense. Controls will be assigned to cases matching on age (within 2 years) and gender. We will aim to assign up to six controls for each case. For inclusion in the nested case–control study, we will restrict to individuals who have been registered with their general practitioner (GP) for at least 1 year prior to the case date (with controls assigned a pseudodate matching the date of outcome for their case) to allow appropriate GP history to construct the health-related variables. For all cases and controls, all health related and comorbidity variables will be calculated relative to the case date. Environmental exposure and metrological variables will be linked based on the appropriate datazones (place of residence, place of work, place of education) for the 3 days prior to the case date, to investigate the time-dependent effects of short-term exposure to air pollution and temperature. We will also use a longer term look back 4–7 days previously, as well as 8–35 days. Average exposure will be calculated for the longer durations. Average as well as maximum exposure will be calculated for the short-term durations. Associations between all covariates (health and environmental) will be measured using conditional logistic regression to account for the matched design.

## Ethics and dissemination

The study received NHS Research Ethics Committee (NHS REC) approval (16/SS/0130). It has also obtained permissions via the Public Benefit and Privacy Panel for Health and Social Care (PBPP) in Scotland to access, collate and use the following data sets: population and housing census for Scotland, Scottish education data via the Scottish Exchange of Data (ScotXed) and primary care data from general practice Data Custodians. We will conduct all data linkage, management and analysis using the secured environment provided by the Scottish Government’s electronic Data Research and Innovation Service (eDRIS) hosted at the Farr Institute in Scotland. The standard protocols provided by eDRIS will be adopted for our data management.

The study protocol will be registered with the European Union electronic Register of Post-Authorisation Studies (EU PAS Register) as a non-interventional post-authorisation study (PAS) before the commencement of data analysis. The results of this study will be published in international peer-reviewed journals. We shall also aim to publish our analytic codes in the open source GitHub website (https://github.com/asthmalhs).

A dissemination workshop will be held in which key stakeholders will be invited to participate. These stakeholders include patients with asthma and representative patient group (PPI), clinicians, leading researchers, policymakers and industry. The meeting will aim to leverage support from affiliated organisations and from individuals who are experts in this area of research to help chart new research lines that will extend our pilot study.

In order to further disseminate our research outputs to a wider audience, we will make presentations at international conferences and local workshops. Media channels of the University of Edinburgh (the Usher Institute Newsletter), Health Data Research UK (eg, The Farr Scotland Newsletter) and the AUKCAR will be used to share publications resulting from this study. We will also engage with policymakers by participating in organised events and meetings where we can showcase the outputs (findings and publications) of this study and interact with policymakers and the general public.

### Data statement

The technical appendix and the data set used for this study will be made accessible via the eDRIS secured platform under the project number: 1516–0489.

## Conclusions

Findings from this project will aid the future development of predictive algorithms for asthma outcomes and can be used to form the basis for our learning health system.

## Supplementary Material

Reviewer comments

Author's manuscript
