# Supplementary material for: Improving predictive asthma algorithms with modelled environment data for Scotland: an observational cohort study protocol
Source: BMJ Open. 2018 May 20;8(5):e023289. doi: 10.1136/bmjopen-2018-023289 (PMC5961591; doi:10.1136/bmjopen-2018-023289)
Supplement: Reviewer comments [file bmjopen-2018-023289.reviewer_comments.pdf]

PEER REVIEW HISTORY

BMJ Open publishes all reviews undertaken for accepted manuscripts. Reviewers are asked to complete a checklist review form (**http://bmjopen.bmj.com/site/about/resources/checklist.pdf**) and are provided with free text boxes to elaborate on their assessment. These free text comments are reproduced below.

ARTICLE DETAILS

**TITLE (PROVISIONAL)**	Improving Predictive Asthma Algorithms with Modelled Environment Data for Scotland: an observational cohort study protocol
**AUTHORS**	Soyiri, Ireneous; Sheikh, Aziz; Reis, Stefan; Kavanagh, Kimberly; Vieno, Massimo; Clemens, Tom; Carnell, Edward; Pan, Jiafeng; King, Abby; Beck, Rachel; Ward, Hester; Dibben, Chris; Robertson, Chris; Simpson, Colin

This article was not externally reviewed at BMJ Open. Protocols that have been independently assessed prior to submission to BMJ Open are usually fast-tracked to publication on the grounds that further substantial changes will not be possible. This independent assessment will usually be external, independent review for both a substantial grant award from a non-commercial or government funder and ethics approval.
